# Cognition, Depression, and Genetics: Examining Sex Differences Using Polygenic and Genetic Inference Techniques

**DOI:** 10.1093/geroni/igaa057.807

**Published:** 2020-12-16

**Authors:** Erin Ware, Arianna Gard, Mingzhou Fu, Lauren Schmitz, Kelly Bakulski, Erin Bakshis Ware

**Affiliations:** 1 University of Michigan, Ann Arbor, Michigan, United States; 2 University of Wisconsin, Madison, Wisconsin, United States

## Abstract

Alzheimer’s disease and its related dementias (ADRD) are debilitating neurodegenerative diseases. As nearly two-thirds of persons diagnosed with Alzheimer’s disease are women, more research is needed to understand sex differences in the biological mechanisms that underlie ADRD. Depression is a risk factor for Alzheimer’s disease and higher rates of depression among women, compared to men, suggest that depression-related phenotypes and underlying biological factors may contribute to sex differences in ADRD. Using the Health and Retirement Study (N = 9908, European ancestry), a US panel-cohort study, the current analysis leverages Mendelian randomization techniques to assess sex-specific inferred causality of depressive symptoms on odds of dementia. All analyses assess most recent cognition and account for sex, education, study cohort, age and year of most recent cognition visit, and genetic ancestry principal components. A one standard deviation increase in depressive polygenic score was associated with 1.11 times higher odds of dementia (95% confidence interval: 1.02-1.21) relative to normal cognition. Each additional endorsed depressive symptom was associated with 1.13 times higher odds of dementia (95% confidence interval: 1.09-1.18) relative to normal cognition. Using the depression genetic instrument, a significant inferred causal relationship was observed between depressive symptoms and dementia (P=0.01, 1.73 odds ratio, 95% confidence interval: 1.12-2.67). When stratified by sex, this relationship was only significant in females (P=0.02, 1.76 odds ratio, 95% confidence interval: 1.08-2.87). These findings demonstrate that depressive symptoms are likely causally related to dementia, and this relationship is most pronounced in females.

